# Pain, Anger, and Rumination in Fibromyalgia: A Vicious Cycle?

**DOI:** 10.3390/jcm14113662

**Published:** 2025-05-23

**Authors:** Michael Tenti, Giorgia Varallo, Federica Cilenti, William Raffaeli, Maristella Scorza, Sandro Rubichi, Giada Pietrabissa, Gianluca Castelnuovo, Paola Gremigni, Giulia Casu

**Affiliations:** 1 ISAL Foundation, Institute for Research on Pain, 47921 Rimini, Italy; michael.tenti@fondazioneisal.it (M.T.); federica.cilenti@fondazioneisal.it (F.C.); william.raffaeli@fondazioneisal.it (W.R.); 2 Studi Cognitivi, Cognitive Psychotherapy School and Research Center, 20121 Milan, Italy; 3 Department of Biomedical, Metabolic and Neural Sciences, University of Modena and Reggio Emilia, 41121 Modena, Italy; g.varallo@unimore.it (G.V.); maristella.scorza@unimore.it (M.S.); sandro.rubichi@unimore.it (S.R.); 4 Psychology Research Laboratory, IRCCS Istituto Auxologico Italiano, 20123 Milan, Italy; 5 Department of Psychology, Università Cattolica del Sacro Cuore, 20123 Milan, Italy; 6 Department of Psychology, University of Bologna, 40127 Bologna, Italy; paola.gremigni2@unibo.it (P.G.); giulia.casu3@unibo.it (G.C.)

**Keywords:** fibromyalgia, pain intensity, metacognitive beliefs, anger rumination, anger, mediation

## Abstract

**Background/Objectives:** Fibromyalgia is a debilitating syndrome characterized by persistent pain. Emerging evidence highlights the role of emotional and cognitive processes in modulating pain experience. Anger, for example, can influence pain and patients’ adjustment to the disease. Studies showed that metacognitions and anger rumination can worsen state anger, thereby increasing perceived pain intensity. The present study aims to investigate the presence of a relationship between pain, dysfunctional metacognitions, anger rumination, and state anger. **Methods:** The study included 446 participants who self-reported having a diagnosis of fibromyalgia confirmed by a rheumatologist or pain specialist. Participants completed self-report measures of metacognitions, anger rumination, state anger, and pain intensity. The serial mediation analysis was performed using Hayes’ PROCESS macro (Model 6). **Results:** Pain intensity showed a significant positive effect on negative beliefs about worry (β = 0.09; *p* < 0.05), need to control thoughts (β = 0.09; *p* < 0.05), and cognitive self-consciousness (β = 0.12; *p* < 0.05), but not on anger rumination. Across all serial mediation models, the direct effect of pain intensity on state anger remained significant even after controlling for the effect of mediators, indicating partial mediation. **Conclusions:** This study suggests a vicious cycle involving dysfunctional metacognitions, anger rumination, state anger, and pain intensity. Our findings also indicate a potential influence of pain on metacognitions and suggest a direct association between pain intensity and state anger. Interventions for anger management in fibromyalgia should consider dysfunctional metacognitions and anger rumination.

## 1. Introduction

Chronic pain, defined as pain that persists or recurs for more than 3 months [1], is a global public health priority. It affects approximately 20% of the general population in Western countries, placing a significant burden on affected individuals, their families, healthcare systems, and society at large [2,3]. The term “chronic pain” encompasses different clinical syndromes [4], which have been recently categorized in the 11th revision of the International Classification of Diseases (ICD-11) [1]. The ICD-11 distinguishes between primary and secondary chronic pain: the former occurs without an identifiable underlying condition, whereas the latter is linked to other diseases as its primary cause [5]. Among chronic primary pain syndromes, fibromyalgia is one of the most challenging conditions for patients to adapt to [6]. It affects 0.2–6.6% of the general population, with a higher prevalence among middle-aged women [7]. Fibromyalgia is characterized by widespread pain persisting for more than 3 months, often accompanied by chronic fatigue, sleep disturbances, cognitive dysfunction, and significant emotional distress. These factors can negatively impact the prognosis of fibromyalgia, increase associated disability, and hinder treatment outcomes [8,9,10,11]. Among the different manifestations of emotional distress, anger is particularly prevalent in fibromyalgia. Sayar and colleagues found that individuals with fibromyalgia experience higher levels of anger than both healthy individuals and patients with other chronic pain conditions [12]. Of note, a recent meta-analysis found a stronger association between pain intensity and state anger than with trait anger [13]. Interestingly, Scarpina et al. found that patients with fibromyalgia presented a lower level of accuracy in recognizing facial expressions of anger when compared to pain-free controls, highlighting potential implicit impairment in dealing with this specific emotion [14]. Patients with chronic pain frequently encounter persistent somatic complaints, diagnostic ambiguity, and recurrent treatment failures, factors that contribute to emotional distress and a heightened risk of anger [15]. Anger may arise from a range of perceived injustices or frustrations directed toward multiple sources, including healthcare professionals, the legal and insurance systems, employers, and significant others [16]. The chronicity and unpredictability of pain, coupled with perceived psychogenic attributions and societal invalidation, may exacerbate feelings of helplessness and alienation, fueling anger [17,18]. Importantly, the presence of a heightened state anger has been consistently associated with greater functional disability and elevated emotional distress, contributing to a maladaptive cycle that perpetuates suffering [13].

**Figure 1:**
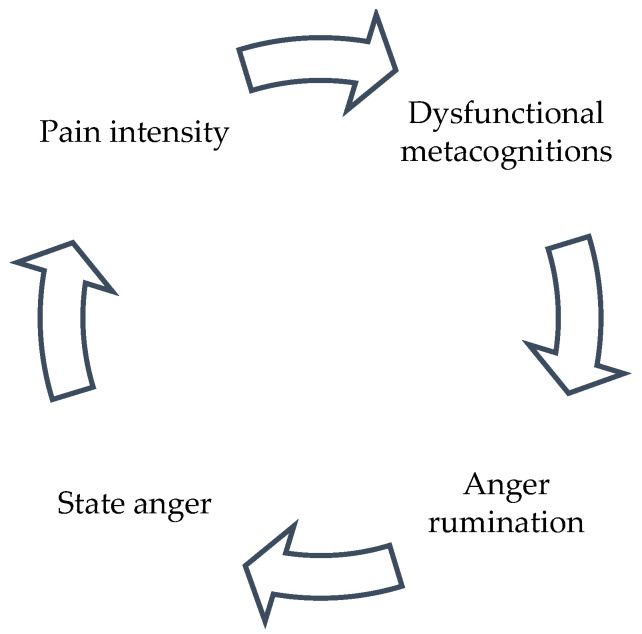
Possible vicious cycle of pain intensity, dysfunctional metacognitions, anger rumination, and state anger.

According to the Self-Regulatory Executive Function (S-REF) model of emotional disorders [19], individuals’ beliefs about their thinking processes and coping strategies, referred to as “metacognitions”, play a key role in activating and maintaining negative emotions such as anger [20]. The S-REF model proposes that, under conditions of stress or threat, dysfunctional metacognitions can trigger maladaptive coping strategies, such as rumination (i.e., a repetitive and passive focus on symptoms of distress and the possible causes and consequences of those symptoms), that sustain emotional disturbances and impair effective emotion regulation. Recent research suggests that dysfunctional metacognitions and anger rumination (i.e., the tendency to repetitively think about frustrating experiences and episodes of anger) negatively affect state anger [21]. Pain may act as a stressor, activating dysfunctional metacognitions that, in turn, trigger anger rumination [22]. This process may result in a heightened state anger, potentially establishing a self-perpetuating cycle (Figure 1).

Considering this interaction, it is important to investigate how these mechanisms function in patients with fibromyalgia. Consequently, the current study seeks to determine whether dysfunctional metacognitions and anger rumination serially mediate the relationship between pain intensity and state anger.

## 2. Materials and Methods

This study is a secondary data analysis from a cross-sectional study conducted on a convenience-based online sample of adults with fibromyalgia. Participants were recruited between July 2021 and October 2021 through the Facebook page of the Italian fibromyalgia patients’ association “Comitato Fibromialgici Uniti-Italia Odv”. The inclusion criteria required the participant to (a) be 18 years or older; (b) self-report a diagnosis of fibromyalgia provided by a rheumatologist or pain physician. After reading the study aims and procedure, participants provided informed consent. The study was approved by the Research Ethics Committee of the Sigmund Freud University of Vienna (Ref. FC29KP4EB18JO388700).

### 2.1. Measures

The online survey collected information on sociodemographics pain duration, the time lag between symptom onset and fibromyalgia diagnosis, the type of specialist who provided the diagnosis (rheumatologist or pain physician), and the perceived effectiveness of current treatments (0 = completely effective to 4 = not at all effective). Pain intensity over the past seven days was measured using the 11-point Numerical Rating Scale (NRS) (0 = no pain to 10 = extreme pain) from the Italian version of the Fibromyalgia Impact Questionnaire—Revised (FIQ-R) [23]. Metacognitive beliefs were assessed using the Italian version of the Short Form Metacognition Questionnaire (MCQ-30) [24]. The MCQ-30 includes 30 items grouped into five subscales: positive beliefs about worry (the belief that worrying is useful), negative beliefs about worry (the belief that worrying is uncontrollable and dangerous), cognitive confidence (confidence in one’s attention and memory), need to control thoughts (the belief that certain thoughts must be suppressed), and cognitive self-consciousness (the tendency to monitor thoughts and focus attention inward). The response scale is a 4-point Likert scale (1 = disagree; 4 = completely agree), and higher scores indicate greater dysfunctionality of the examined metacognitive beliefs. In the current study, internal consistency was α = 0.85 for the subscale “positive beliefs about worry” α = 0.70 for “negative beliefs about worry” α = 0.90 for “cognitive confidence” α = 0.67 for “need to control thoughts”, and α = 0.70 for “cognitive self-consciousness”. Anger rumination was measured using the Italian version of the Anger Rumination Scale (ARS) [25], a 13-item self-report questionnaire assessing an individual’s tendency to focus attention on and ruminate about anger episodes. The response scale is a 4-point Likert scale (1 = almost never; 4 = almost always), and higher scores indicate greater anger rumination. ARS does not have any subscales in its Italian version, and, in the present study, the internal consistency was α = 0.94. State anger was assessed using the 10-item state anger subscale of the Italian version of the State–Trait Anger Expression Inventory (STAXI-SA) [26]. The response scale for the items is a 4-point Likert scale (1 = not at all; 4 = very much), and higher scores indicate greater state anger. In the present study, internal consistency was α = 0.93.

### 2.2. Data Analysis

Participant demographics and pain characteristics were summarized using means (SD) or frequencies, as appropriate. Preliminary analyses were conducted to assess zero-order correlations among study variables and identify potential covariates to be controlled for in the mediation model. Covariate selection involved examining the correlations of the mediator and outcome variables with age, pain duration, time lag between symptom onset and fibromyalgia diagnosis, and perceived efficacy of current treatments. Variables showing a significant correlation with either the mediators or the outcome with at least a medium effect size (r ≥ |0.30|) were included as covariates [27].

The serial mediation of metacognitions and anger rumination in the relationship between pain intensity and state anger was tested in IBM SPSS 25 using PROCESS (model 6). Statistical significance was set at α = 0.05. The significance of indirect effects was evaluated using bootstrapped confidence intervals (CIs), with CIs that did not include zero considered statistically significant.

## 3. Results

Of the 504 participants who completed the online survey, 446 (88.5%) reported having received a fibromyalgia diagnosis from a rheumatologist or pain physician and were included in the study based on eligibility criteria and completed all the measures. Their mean age was 49.36 years (SD = 10.18, range 23–77 years), with 97.3% identifying as female. More than half of the participants (51.8%) had been experiencing chronic pain for over ten years, while in nearly half (48%), fibromyalgia was diagnosed within three years of symptom onset. A total of 17.7% of participants reported not using any medication or treatment for fibromyalgia. Only a small proportion described their treatment as “fully effective” (0.4%) or “very effective” (5.6%), while 16.4% found their therapies completely ineffective. A detailed description of the sample is available in the original study [21].

As reported elsewhere [21], preliminary correlation analyses showed that anger rumination and state anger were both positively correlated with pain intensity. Only negative beliefs about worry, the need to control thoughts, and cognitive self-consciousness were significantly correlated with anger rumination, state anger, and pain intensity; therefore, only these dysfunctional metacognitions were included in the serial mediation model. State anger was not significantly associated with age (r = 0.04, *p* > 0.05), pain duration (r = 0.05, *p* > 0.05), time lag between symptom onset and fibromyalgia diagnosis (r = 0.04, *p* > 0.05), or perceived efficacy of current treatments (r = 0.04, *p* > 0.05). Negative beliefs about worry, need to control thoughts, and cognitive self-consciousness were not significantly associated with pain duration (respectively: r = −0.01, *p* > 0.05; r = 0.00, *p* > 0.05; r = −0.08; *p* > 0.05), time lag between symptom onset and fibromyalgia diagnosis (respectively: r = −0.03, *p* > 0.05; r = −0.04, *p* > 0.05; r = −0.02; *p* > 0.05), or perceived efficacy of current treatments (respectively: r = −0.07, *p* > 0.05; r = −0.05, *p* > 0.05; r = −0.01; *p* > 0.05). Cognitive self-consciousness, but not negative beliefs about worry nor need to control thought, presented a significant weak association with age (respectively: r = −0.19, *p* < 0.001; r = −0.03, *p* > 0.05; r = −0.01; *p* > 0.05). Consequently, no covariates were included in the serial mediation analysis.

In the mediation analyses, higher pain intensity was directly associated with higher state anger. Regarding specific indirect effects, pain intensity had significant specific indirect effects on anger rumination through negative beliefs about worry, need to control thoughts, and cognitive self-consciousness. Higher pain intensity was linked to higher levels of negative beliefs about worry and cognitive self-consciousness, which, in turn, were associated with higher state anger. Additionally, higher pain intensity was related to greater cognitive self-consciousness, which, in contrast, was associated with lower state anger. The specific indirect effects of pain intensity via anger rumination were nonsignificant, and the effect of pain intensity on anger rumination was also nonsignificant. However, all serial indirect effects were significant: higher pain intensity was sequentially associated with higher levels of negative beliefs about worry, need to control thoughts, and cognitive self-consciousness, which then led to higher anger rumination, ultimately resulting in increased state anger. The complete regression-based analysis results are shown in Table 1.

**Figure 2:**
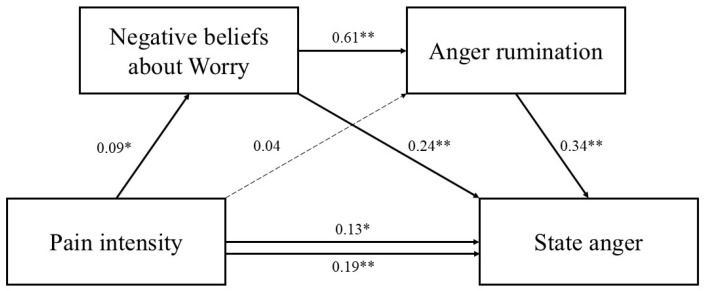
Serial mediation model including negative beliefs about worry as the first mediator. Standardized parameter estimates are reported. Dashed lines indicate nonsignificant paths. * *p* < 0.05. ** *p* < 0.001.

**Figure 3:**
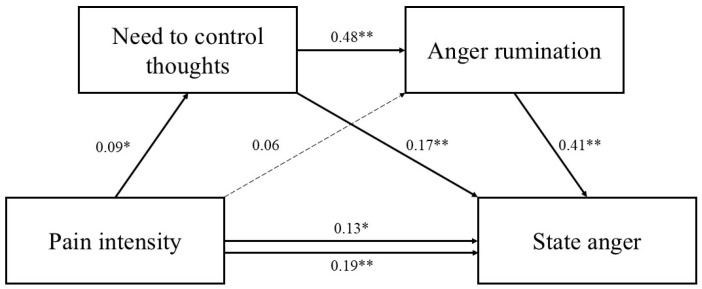
Serial mediation model including need to control thoughts as the first mediator. Standardized parameter estimates are reported. Dashed lines indicate nonsignificant paths. * *p* < 0.05. ** *p* < 0.001.

**Figure 4:**
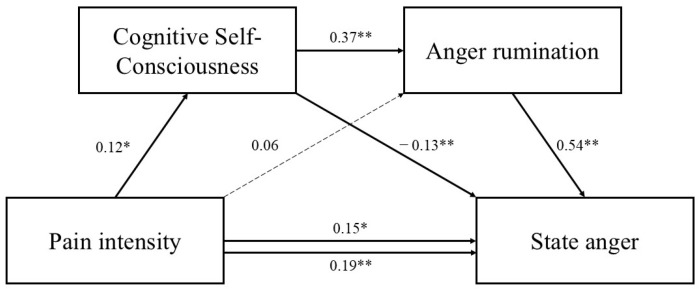
Serial mediation model including cognitive self-consciousness as the first mediator. Standardized parameter estimates are reported. Dashed lines indicate nonsignificant paths. * *p* < 0.05. ** *p* < 0.001.

Standardized path coefficients are presented in Figure 2, Figure 3 and Figure 4.

The total, specific, and serial indirect effects are shown in Table 2.

**Figure 5:**
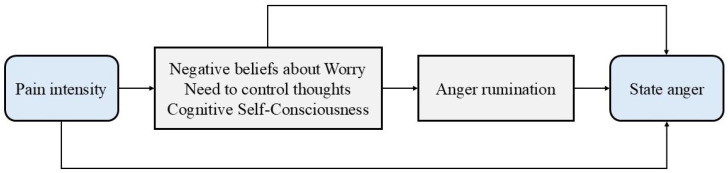
Graphical summary of serial mediation paths examined in the study.

Figure 5 provides a graphical summary of the serial mediation pathways examined in the study.

## 4. Discussion

Previous research indicates that, in patients with fibromyalgia, dysfunctional metacognitions and anger rumination negatively affect state anger, which, in turn, may exacerbate pain intensity [21]. This study expands on these findings by showing that pain intensity may contribute to anger both directly and indirectly through dysfunctional metacognitions and anger rumination. This highlights a potential vicious cycle in which pain intensity, dysfunctional metacognitions, anger rumination, and state anger interact in a self-reinforcing loop. Our findings align with the existing literature on the role of dysfunctional metacognitions (especially negative beliefs about worry and need to control thoughts) in anger rumination [28]. Additionally, our findings provide preliminary evidence that pain may shape specific metacognitions, such as negative beliefs about worry, the need to control thoughts, and cognitive self-consciousness.

The effect of pain on negative beliefs about worry may stem from the fact that chronic pain, as a persistent experience, can repeatedly trigger intrusive thoughts, leading to the belief that rumination is inevitable and uncontrollable. When individuals become aware of the negative impact of these pain-related thoughts, they may attempt to suppress them. However, suppression strategies are often counterproductive: efforts to control one’s thoughts can paradoxically increase their prominence [29], thereby intensifying rumination and anger. Additionally, the frustration arising from unsuccessful suppression attempts may directly contribute to heightened state anger [28].

For what concerns cognitive self-consciousness, the persistence of pain over time may promote an attentional bias toward one’s internal experiences, both physical and cognitive, leading to heightened self-conscious awareness. This is consistent with evidence from multiple studies investigating selective attention to pain-related stimuli in individuals with chronic pain. In their meta-analysis of studies using the modified Stroop paradigm, Roelofs et al. found that chronic pain patients selectively attend to pain-related sensory and affective information [30]. More recently, Todd et al., in a meta-analysis of dot-probe studies, identified a small but significant attentional bias toward sensory pain words in both acute and chronic pain patients, but not in those anticipating pain or in healthy individuals [31]. This heightened cognitive self-focus may not only amplify pain-related thoughts but also increase the salience of other internally generated cognitions, including anger-related ones. As a result, individuals may become more prone to ruminate on anger, further reinforcing maladaptive cognitive patterns that contribute to emotional distress [21].

Interestingly, although a positive correlation between pain intensity and anger rumination emerged in preliminary analyses, in the mediation model, both the indirect effects of pain intensity on state anger via anger rumination and the direct effect of pain intensity on anger rumination were nonsignificant. These findings suggest that pain intensity may not directly trigger anger rumination but affects it indirectly through the mediation effect of specific metacognitive beliefs. This would be in line with the metacognitive model [19] and would emphasize the central role of metacognitions in the emotional consequences of pain. On the other hand, the above-mentioned result of the mediation model may be due to the strength of the preliminary correlations, which might not be sufficient to create a significant mediated effect.

Our study also offers insights into the relationship between pain intensity and state anger. While this relationship is often described in the literature as bidirectional, few studies have focused on the effect of pain on anger [13]. Our findings suggest a direct effect of pain intensity on state anger. Different factors may explain this relationship. For instance, Trost et al. hypothesized that anger in the context of chronic pain may be influenced by key cognitive factors such as perceived injustice and external attribution [32]. Individuals with chronic pain may perceive themselves as victims of injustice or blame others for their pain. Over time, this perceived injustice and externalization of blame may intensify feelings of anger, potentially worsening disability and making pain resistant to conventional treatments [33]. In summary, the relationship between chronic pain and anger is complex and multifaced, with potential mediators not considered in this study, such as perceived injustice and external blame attribution.

This study has important clinical implications. Although interventions for anger management in fibromyalgia should consider the influence of dysfunctional metacognitions and anger rumination, the effects of pain on metacognitions highlight the importance of validating patients’ pain experiences and their impact on metacognitions. Metacognitive therapy interventions could benefit from incorporating a psycho-educational component into the therapeutic plan. This addition would help patients understand how pain shapes metacognitive beliefs while also addressing the detrimental effects of dysfunctional metacognitions on emotional distress and pain perception.

This study has some limitations. First, its cross-sectional design restricts the ability to infer causality in the proposed models. Future research should also explore the bidirectional interplay between pain and anger, considering additional cognitive and emotional mediators such as perceived injustice and external blame attribution. Longitudinal studies and experimental designs would help clarify causal relationships and refine targeted interventions. Second, while the MCQ-30 [24] is a validated tool for assessing metacognitive beliefs related to worry, it may be less specific for those related to anger rumination. Unfortunately, no validated tool on anger rumination-related metacognitions is currently available in Italian. Future studies could address this gap by developing or adapting such a tool. The present study relies on self-reported fibromyalgia diagnosis, without clinical verification; this could limit the generalizability of our findings to populations with confirmed diagnoses. A further limitation of the study is the lack of assessment and statistical control for age at onset and relevant comorbidities, such as depression, anxiety, and sleep disorders, which are known to influence both pain processing and emotional regulation [34,35].

Despite these limitations, the findings of the present study—together with previous literature on the topic—highlight a significant relationship between pain, metacognitive beliefs, anger rumination, and anger, paving the way for future investigations into these underexplored processes in the context of chronic pain and fibromyalgia.

## Figures and Tables

**Table 1:** Regression-based results in serial mediation models.

Criterion	Predictors	R	R^2^	F	B	t	95% CIs
Negative beliefs about worry	Pain intensity	0.10	0.01	4.61	0.21	2.15	[0.018, 0.405]
Anger rumination	Pain intensity	0.62	0.38	137.25	0.23	1.19	[−0.153, 0.622]
	Negative beliefs about worry				1.55	16.32	[1.36, 1.73]
State anger	Pain intensity	0.56	0.31	65.72	0.56	3.28	[0.223, 0.890]
	Negative beliefs about worry				0.49	4.78	[0.290, 0.695]
	Anger rumination				0.28	6.76	[0.196, 0.357]
Need to control thoughts	Pain intensity	0.09	0.01	3.79	0.17	1.95	[−0.002, 0.349]
Anger rumination	Pain intensity	0.49	0.24	68.55	0.33	1.51	[−0.101, 0.761]
	Need to control thoughts				1.33	11.42	[1.101, 1.559]
State anger	Pain intensity	0.54	0.29	61.85	0.56	3.29	[0.227, 0.899]
	Need to control thoughts				0.39	3.80	[0.190, 0.595]
	Anger rumination				0.33	8.87	[0.256, 0.402]
Cognitive self-consciousness	Pain intensity	0.12	0.01	6.29	0.23	2.51	[0.050, 0.411]
Anger rumination	Pain intensity	0.38	0.14	37.04	0.33	1.43	[−0.124, 0.792]
	Cognitive self-consciousness				0.99	8.26	[0.754, 1.225]
State anger	Pain intensity	0.54	0.29	59.04	0.64	3.68	[0.296, 0.975]
	Cognitive self-consciousness				−0.28	−2.89	[−0.462, −0.088]
	Anger rumination				0.43	12.30	[0.364, 0.503]

**Table 2:** Total, direct, and serial indirect effects in the mediation models.

Effect	B	SE(B)	95% CIs	β	SE(β)	95% CIs
**Negative beliefs about worry**						
Total effect	0.816	0.198	[0.425, 1.205]	0.19		
Direct effect	0.557	0.169	[0.223, 0.890]	0.13		
Total indirect effect	0.259	0.105	[0.058, 0.472]	0.06	0.02	[0.014, 0.109]
***Specific indirect effects***						
Pain → Negative beliefs about worry → State anger	0.104	0.056	[0.007, 0.229]	0.02	0.01	[0.002, 0.053]
Pain → Anger rumination → State anger	0.065	0.052	[−0.039, 0.169]	0.02	0.01	[−0.009, 0.039]
Serial indirect effect	0.090	0.046	[0.006, 0.190]	0.02	0.01	[0.001, 0.044]
**Need to control thoughts**						
Total effect	0.816	0.198	[0.426, 1.206]	0.19		
Direct effect	0.563	0.171	[0.227, 0.900]	0.13		
Total indirect effect	0.252	0.100	[0.061, 0.456]	0.06	0.02	[0.015, 0.106]
***Specific indirect effects***						
Pain → Need to control thoughts → State anger	0.068	0.041	[0.001, 0.162]	0.02	0.01	[0.000, 0.038]
Pain → Anger rumination → State anger	0.109	0.069	[−0.026, 0.246]	0.03	0.02	[−0.006, 0.057]
Serial indirect effect	0.076	0.040	[0.002, 0.156]	0.02	0.01	[0.000, 0.037]
**Cognitive self-consciousness**						
Total effect	0.816	0.198	[0.426, 1.206]	0.19		
Direct effect	0.663	0.173	[0.296, 0.975]	0.15		
Total indirect effect	0.180	0.096	[−0.006, 0.367]	0.04	0.02	[−0.001, 0.085]
***Specific indirect effects***						
Pain → Cognitive self-consciousness → State anger	−0.063	0.035	[−0.141, −0.007]	−0.01	0.01	[−0.033, −0.002]
Pain → Anger rumination → State anger	0.145	0.094	[−0.039, 0.331]	0.03	0.02	[−0.009, 0.076]
Serial indirect effect	0.099	0.042	[0.021, 0.184]	0.02	0.01	[0.005, 0.044]

SE = standard error; CI = confidence interval.

## Data Availability

The data presented in this study are available on reasonable request from the corresponding author.

## Abbreviations

The following abbreviations are used in this manuscript:

ARS	Anger Rumination Scale
CC	Cognitive confidence
CIs	Confidence intervals
CSC	Cognitive self-consciousness
FIQ-R	Fibromyalgia Impact Questionnaire—Revised
ICD-11	International Classification of Diseases-11
MCQ-30	Short Form Metacognition Questionnaire
NC	Need to control thoughts
NEG	Negative beliefs about worry
NRS	Numerical Rating Scale
POS	Positive beliefs about worry
SE	Standard error
S-REF	Self-Regulatory Executive Function
STAXI-SA	State–Trait Anger Expression Inventory
